# Lifestyle Factors Associated with Undernutrition in Older People after the Great East Japan Earthquake: A Prospective Study in the Fukushima Health Management Survey

**DOI:** 10.3390/ijerph19063399

**Published:** 2022-03-14

**Authors:** Kanako Okazaki, Tetsuya Ohira, Akira Sakai, Michio Shimabukuro, Junichiro J. Kazama, Atsushi Takahashi, Hironori Nakano, Fumikazu Hayashi, Masanori Nagao, Seiji Yasumura, Hitoshi Ohto, Kenji Kamiya

**Affiliations:** 1 Department of Physical Therapy, Fukushima Medical University School of Medical Sciences, Fukushima 960-8516, Japan; 2 Radiation Medical Science Center for the Fukushima Health Management Survey, Fukushima Medical University, Fukushima 960-1295, Japan; teoohira@fmu.ac.jp (T.O.); sakira@fmu.ac.jp (A.S.); shima01@fmu.ac.jp (M.S.); jjkaz@fmu.ac.jp (J.J.K.); junior@fmu.ac.jp (A.T.); h-nakano@fmu.ac.jp (H.N.); fhayashi@fmu.ac.jp (F.H.); nagaom@fmu.ac.jp (M.N.); yasumura@fmu.ac.jp (S.Y.); hit-ohto@fmu.ac.jp (H.O.); kkamiya@fmu.ac.jp (K.K.); 3 Department of Epidemiology, Fukushima Medical University School of Medicine, Fukushima 960-1295, Japan; 4 Department of Radiation Life Sciences, Fukushima Medical University School of Medicine, Fukushima 960-1295, Japan; 5 Department of Diabetes, Endocrinology and Metabolism, Fukushima Medical University School of Medicine, Fukushima 960-1295, Japan; 6 Department of Nephrology and Hypertension, Fukushima Medical University School of Medicine, Fukushima 960-1295, Japan; 7 Department of Gastroenterology, Fukushima Medical University School of Medicine, Fukushima 960-1295, Japan; 8 Department of Public Health, Fukushima Medical University School of Medicine, Fukushima 960-1295, Japan; 9 Research Institute for Radiation Biology and Medicine, Hiroshima University, Hiroshima 734-8553, Japan

**Keywords:** older people, undernutrition, the Great East Japan earthquake, lifestyle factors, regular exercise, physical activity

## Abstract

We conducted a longitudinal examination to assess the relationship between lifestyle habits, including exercise habits, and the incidence of undernutrition after the Great East Japan Earthquake in March 2011. Of the 31,411 participants aged ≥60 years who lived in the municipalities’ evacuation areas before the disaster and had undergone health examinations, 17,622 persons with a body mass index of 20–25 kg/m^2^ were followed up through the FY 2017 (a mean follow-up of 6.9 years). The analysis involved 13,378 individuals who could be followed. The associations between undernutrition after the disaster and lifestyle factors were estimated via multivariable-adjusted analysis using the Cox proportional hazard regression model. The dependent variable was the proportion of undernutrition after the disaster, whereas independent variables included evacuation, exercise habits/physical activity, alcohol consumption, smoking, meals before bedtime, gastrointestinal surgery history, history of lifestyle-related diseases, and two or more subjective symptoms. In total, 1712 of the 13,378 participants were newly undernourished after the disaster. The statistically significant variables influencing the occurrence of undernutrition were non-evacuation (hazard ratio (HR), 1.31; 95% confidence index (CI) 1.17–1.47), poor exercise habits (HR, 1.14; 95% CI 1.03–1.50), and poor physical activity (HR, 1.12; 95% CI 1.01–1.25). Other significant related variables were drinking habits, surgical history, lifestyle-related diseases, and two or more subjective symptoms. These results suggest that regular exercise and/or physical activity might be important in preventing undernutrition following a disaster, regardless of sex, other lifestyle habits, or past medical history.

## 1. Introduction

With the increasing age of the Japanese population, extending the healthy life expectancy is a problem. Reportedly, 70% of men and 90% of women in older adults aged 60 years old or more are less independent due to a dysfunction caused by the geriatric syndrome. To maintain the functional independence of the elderly, not only prevention of disease onset but also prevention of geriatric syndrome caused by aging change, are essential issues [1]. Older individuals tend to have a low physical activity, reduced appetite, and low nutrient intake. This results in undernutrition and reduced muscle mass, which in turn lowers the baseline metabolism and limb muscle strength, thus causing reduced walking speed, greater difficulties in getting around, and lower activity. This situation is known as “the frailty cycle” [2,3]. A survey of 5104 people aged 65 years and older (mean age at onset, 71 years old) was conducted in 2011–2012 in Japan. The result showed that 11.3% fell into the category of frailty, according to the Cardiovascular Health Study criteria [4]. The analysis results that integrated four representative large-scale cohort studies using these criteria in Japan indicated that the frailty prevalence in the community-dwelling older population was 7.4%. The frailty prevalence increases with age and markedly increases after 75 years [5]. In contrast, according to the National Health and Nutrition Survey conducted annually by the government, the percentage of undernourishment (BMI ≤ 20 kg/m^2^) of those aged 65 years or older was 16.4% in 2017. In addition, about 20% of both men and women aged 80 years or older tend to be undernourished [6], which is a major risk factor for health deterioration. Early detection and intervention for undernutrition and decreased activity are important for interrupting this vicious circle. However, there are no studies that showed the actual weight loss and undernutrition status in the evacuation areas after the disaster and the relationship between lifestyle and undernutrition due to evacuation after the disaster.

After the Great East Japan Earthquake (GEJE), on 11 March 2011, and the subsequent accident at the Fukushima Daiichi Nuclear Power Plant, evacuees from the government-designated evacuation areas were forced to move and change their lifestyle behaviors, including diet, exercise, and other personal habits. Many evacuees had to change jobs, and some received inadequate health check-ups and experienced varying degrees of anxiety about their health. Following the disaster, these changes in the living environment have resulted in increased psychological stress and decreased physical activity [7,8,9,10,11]. According to the Fukushima Health Management Survey (FHMS) conducted in the Fukushima Prefecture after the disaster, lifestyle-related diseases were confirmed to worsen with increasing body weight [12,13,14,15,16,17]. Some residents, however, lost weight and reduced their nutrition intake due to the disaster. The FHMS reported that people in the evacuation centers, temporary housing, apartments, etc., consumed less fruit and vegetables, meat, and soybean and dairy products than those who lived in their own or relative’s home [18]. Therefore, the frailty risk is expected to increase in the older evacuees due to aging, reduced physical activity, decreased nutrient intake, and changes in the living environment [19].

Frailty requires long-term care and is affected by the damage caused by a disaster [19]. Thus, weight loss and undernourishment thinness (BMI ≤ 20.0 kg/m^2^) in the elderly are essential factors of frailty, and it has been reported that lifestyle is related to weight loss and undernourishment [3].

Therefore, in this study, to investigate the factors associated with the undernutrition onset among older people in the evacuation area after the March 2011 disaster, we used the health examinations results before and after the disaster. Subsequently, we followed them until FY 2017, the year after the disaster, to longitudinally examine the relationship between the undernutrition occurrence and lifestyle factors, such as exercise and dietary behavior. It was hypothesized that individuals who had physical activity and exercise habits before the disaster would have a lower undernutrition risk after the disaster.

## 2. Materials and Methods

### 2.1. Study Population

The participants were residents aged 60 years or more living in evacuation-designated areas near the Fukushima Daiichi Nuclear Power Plant in the Fukushima Prefecture before March 2011, when the earthquake occurred. The evacuation area included 13 municipalities: Hirono-machi, Naraha-machi, Tomioka-machi, Kawauchi-mura, Okuma-machi, Futaba-machi, Namie-machi, Katsurao-mura, Iitate-mura, Kawamata-machi, Tamura City, Minami-Soma City, and Date City. Within the evacuation area, those aged 40–74 years had enrolled in the national health insurance, and those aged 75 years or more had enrolled in the medical system for the elderly and underwent annual health check-ups.

Between FY 2008, before the disaster, and FY 2017, after the disaster, a total of 254,161 individuals aged 60 years or more underwent medical examinations. Of these, 68,010 examinees aged 60 years or more underwent at least one health check-up before the disaster during FY 2008–2010 (baseline period). We excluded the second and subsequent results if there was more than one visit during this period. We, therefore, included 31,411 participants (14,350 men and 17,061 women; mean age, 69.8 years) who underwent a health examination during this period.

**Figure 1:**
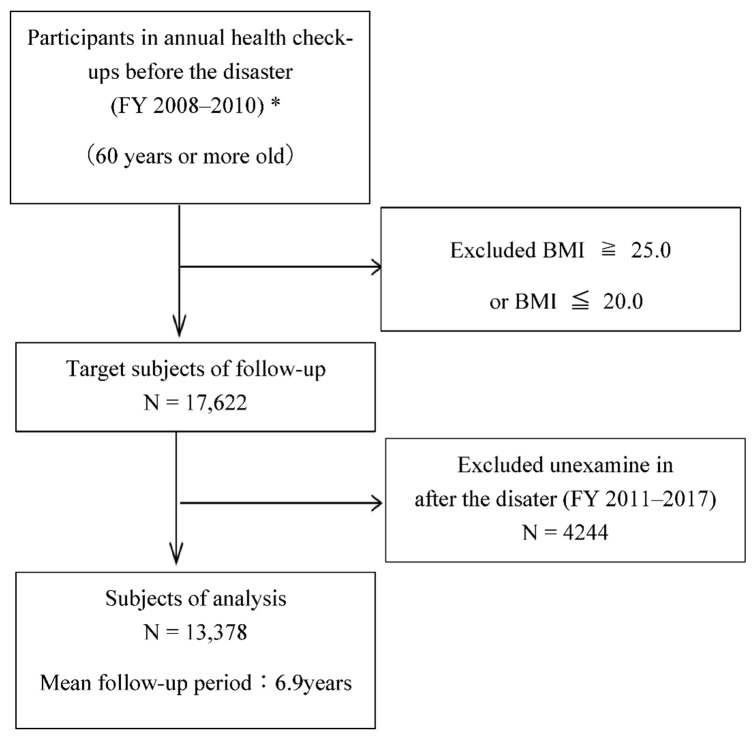
The selection process for the study patients. * In case of duplication, exclude the data of the second and third exams.

We excluded 13,789 individuals who were overweight (BMI ≥ 25.0 kg/m^2^) or undernourished (BMI ≤ 20.0 kg/m^2^); 17,622 were eligible for follow-up examinations that were conducted from FY 2011–FY 2017 after the disaster. Given that 4244 participants did not undergo follow-up examinations, 13,378 (6351 men and 7027 women, 76%) were ultimately eligible for our analysis (Figure 1).

### 2.2. Measurements/Definitions and Data Collection

The pre-disaster data were provided by the medical examinations conducted by municipalities and included specific health examinations and late-elderly health examinations. For the post-disaster follow-up data, the FHMS and the above data obtained from the municipalities were used. The baseline and follow-up examinations included a medical history review, physical examination, anthropometric measurements, and questionnaire regarding lifestyle behaviors. This study was approved by the Ethics Committee of Fukushima Medical University (#1319, #1916).

#### 2.2.1. Undernutrition

The participants’ body weight and height were measured (with their shoes and excess clothing removed) on the same calibrated scale at baseline and follow-up. BMI was calculated as the body weight (kg) divided by the square of the height (m^2^). According to the National Health and Nutrition Survey, undernutrition was defined as a BMI ≤ 20.0 kg/m^2^. Some researchers recommend using BMI as an objective indicator in older individuals [20,21,22]. BMI is not a sensitive indicator in a clinical setting for showing rigorous changes in clinical situations because individuals with a normal or high BMI can have a clinically significant weight loss. Older individuals often have spinal deformity and difficulty standing; therefore, the height measurement required to calculate BMI is unreliable [23]. However, in this study, the target participant was a community-living older individual who could undergo a health check-up. Some studies have used the BMI measured during the health check-up as an indicator of undernutrition [24]. Therefore, this definition was used.

#### 2.2.2. Weight Loss Amount

In previous studies, the weight loss criterion for determining frailty was “2–3 kg in 6 months.” [25,26]. As an “unintended sudden weight loss” for determining geriatric syndrome risk, there was the index “5% weight loss in 6–12 months” [27]. However, identifying the weight loss within such a short period is challenging using the medical examination data in this study. Therefore, the change and rate of change were calculated throughout the observation period as follows: weight loss (≥5 kg or ≥5%) from baseline; amount of weight loss, kg; weight loss per year, kg/year; weight loss rate (loss/weight at baseline); and weight loss rate per year, %/year.

#### 2.2.3. Lifestyle Status

Information on lifestyle factors, including smoking status, exercise habits, physical activities, insufficient sleep, and dietary behaviors, were obtained from a self-administered questionnaire from the standard interview items during the medical checkups [28] and were classified into two categories. Smoking status was shown as regular smoker or nonsmoker. Insufficient sleep was defined by whether the sleep provided rest (yes/no). Dietary behaviors were defined as follows: skipping breakfast (skipping breakfast more than thrice a week), meals before going to bed (having dinner within two hours before going to bed at least thrice a week) and snacking after dinner (more than three snacks after dinner a week). Drinking status was shown by the frequency of drinking and the amount of alcohol consumed per day and was categorized into three groups for the weekly alcohol intake: Never drinks (including quitting); Drinks < 44 g/day; and Drinks > 44 g/day. Other items in the standard interview were as follows: weight gain >10 kg from 20 years of age (yes/no), change in body weight >3 kg in one year (yes/no), and walking speed (faster walking speed than the same age and sex, yes/no). The “Physical Activity reference for Health Promotion 2013” distinguishes between “exercise habits” for sports and physical fitness and those related to living, such as employment, housework, and mobility [29]. The questionnaire items for the medical examination were set according to these criteria: exercise habit was defined by whether the individual lightly sweated and exercised for at least 30 min at least twice a week for more than one year. Physical activity was defined as walking for one or more hours per day or equivalent activity, such as employment, housework, and mobility.

#### 2.2.4. Medical History

Information regarding medical history, surgery, and subjective symptoms was collected through interviews with local public health nurses. A history of gastrointestinal surgery was defined as at least one esophageal occurrence, stomach, duodenal, or colon surgery. A history of lifestyle-related disease was defined as a history of any of the following conditions: hypertension, dyslipidemia, diabetes, hepatic dysfunction, or renal dysfunction. Subjective symptoms over the past year were classified into three categories: none, one, two or more.

#### 2.2.5. Evacuees

There was no information regarding the individuals’ evacuation status based on their residential area in the target municipalities. All residents in areas designated as evacuation areas were defined as evacuees, and those undesignated as evacuation areas were non-evacuees.

### 2.3. Statistical Analysis

The participants were divided into two groups: undernourished (*n* = 1712) and not undernourished (*n* = 13,019). Participants with and without undernutrition were compared using the chi-square test for categorical variables and the *t*-test for continuous variables. We tested the associations between undernutrition after the disaster and other primary lifestyle factors using a simple, sex/age-adjusted, and multivariate-adjusted analysis using the Cox proportional hazard regression model. Although there were missing values for lifestyle factors, there was no difference in the mean age, BMI, sex distribution, malnutrition, evacuation, exercise habits, and physical activity, even when the missing values were excluded. Therefore, these values were treated as missing, ensuring representativeness. A univariate analysis was performed after deleting the missing data; in the multivariate analysis, the adjustments were made by inserting dummy variables into the missing data. In the multivariate-adjustment model, items associated in a statistically significant manner with undernutrition in the sex-age-adjustment model were employed as adjustment variables. To avoid multicollinearity of exercise habits and physical activity, one of the habits was employed as an adjustment variable if both were statistically significant in a sex-age-adjusted analysis. The condition for censoring was that the follow-up period for undernutrition was defined as the period until the first case of undernutrition was detected. For cases without undernutrition, the condition for censoring was defined as the medical check-up date for the FY 2017 or the final medical check-up date earlier that year. The person years were calculated as the sum of the individual follow-up times until undernutrition incidence or the last examination date.

SAS version 9.4 (SAS Institute, Cary, NC, USA) was employed for all statistical analyses, with two-tailed probability values for the statistical tests. A *p*-value ≤0.05 was considered statistically significant.

## 3. Results

### 3.1. Frequency and Characteristics of Undernutrition after the Disaster

Table 1 indicated the baseline characteristics of the participants with and without undernutrition. Their mean age was 68.4 ± 6.2 years, and 46.6% were men. During the 6.9-year follow-up, 1712 (12.8%) of the participants were undernourished and were significantly older, with a higher percentage of women and a lower percentage of evacuees.

Compared with the participants without undernutrition, the frequency of meals before going to bed, exercise habits, physical activity, smoking habits, and alcohol consumption were lower among those with undernutrition. The proportion of lifestyle-related diseases was significantly higher among those with undernutrition than among those without undernutrition.

### 3.2. Lifestyle Factors Associated with Undernutrition

Table 2 shows the sex/age-adjusted and multivariate-adjusted hazard ratios (HRs) and 95% confidence intervals (CIs) for each lifestyle factor of undernutrition. The multivariate-adjusted model included variables significantly associated with the sex-age-adjustment model. Since exercise habits and physical activity were categorical variables, we calculated it using Cramer’s V and found it to be 0.753, which we judged to be a strong association. Therefore, if both of these factors were statistically significant in the sex-age-adjustment analysis, only one of them was used as an adjustment variable to avoid multicollinearity.

The statistically significant variables influencing the onset of undernutrition in Model 1 were as follows: non-evacuation (HR, 1.31; 95% CI 1.17–1.47), poor (< 30 min/2 times/week) exercise habits (HR, 1.14; 95% CI 1.03–1.27), no/infrequent meals before going to bed (HR, 1.26; 95% CI 1.11–1.43), history of gastrointestinal surgery history (HR, 1.24; 95% CI 1.03–1.50), history of lifestyle-related disease (HR, 1.27; 95% CI 1.16–1.40), and two or more subjective symptoms (HR, 1.26; 95% CI 1.04–1.53). When baseline BMI was added as an adjustment variable in Model 3, the adjusted HR (and 95% CI) of exercise habits remained almost unchanged 1.11 (1.00–1.24; *p* = 0.005). Furthermore, a multiple regression analysis using the change in BMI as the dependent variable and the same adjustment variables as in Model 1 indicated that gender, age, evacuation, smoking, and late dinners were associated with weight loss. Nevertheless, exercise habit was insignificant (Table S1).

The variables that significantly influenced the onset of undernutrition in Model 2 were as follows: non-evacuation (HR, 1.31; 95% CI 1.17–1.47), poor physical activity (HR, 1.14; 95% CI 1.01–1.25), no/infrequent meals before going to bed (HR, 1.25; 95% CI 1.01–1.42), history of gastrointestinal surgery (HR, 1.24; 95% CI 1.02–1.49), history of lifestyle-related disease (HR, 1.28; 95% CI 1.16–1.41), and two or more subjective symptoms (HR, 1.26; 95% CI 1.04–1.52). In contrast, in Model 4, with the baseline BMI added as an adjustment variable, the adjusted HR (and 95% CI) of physical activity was 1.08 (0.98–1.20; *p* = 0.142). Furthermore, in a multiple regression analysis, with the change in BMI as the dependent variable (adjusted variables were the same as in Model 2), gender, age, evacuation, and late supper were shown to be associated with weight loss, while exercise habits were not significant (Table S1).

Table 3 indicated the HRs of exercise habits and physical activity with the incidence of undernutrition after the disaster in the multivariate-adjustment model, stratified by each lifestyle factor. Regarding the results of the stratified analysis, the groups that exhibited a significant association between exercise habits/physical activity and undernutrition in each analysis are as follows. In the exercise habit analysis, a significant association (*p* < 0.05) between exercise habits and undernutrition was found in men (HR, 1.21; 95% CI 1.02–1.44), aged under 75 years (HR, 1.18; 95% CI 1.05–1.33), BMI at baseline <27.0 kg/m^2^ (HR, 1.15; 95% CI 1.03–1.29), non-evacuation group (HR, 1.19; 95% CI 1.05–1.35), smoking group (HR, 1.54; 95% CI 1.10–2.14), moderate drinking group (HR, 1.22; 95% CI 1.02–1.46), and frequent late dinner group (HR, 1.14; 95% CI 1.02–1.28). The interaction with exercise habit was significant only at baseline BMI. In contrast, for physical activity, there was a significant association (*p* < 0.05) between physical activity and undernutrition in women (HR, 1.15; 95% CI 1.01–1.31), those under 75 years old (HR, 1.15; 95% CI 1.02–1.29), non-smoking group (HR, 1.14; 95% CI 1.02–1.27), and non-drinking group (HR, 1.19; 95% CI 1.05–1.36). Additionally, no statistically significant interaction with physical activity was confirmed for each lifestyle factor.

## 4. Discussion

### 4.1. Exercise Habits and Physical Activities

In this study, inadequate exercise habits and physical activities affected undernutrition onset after the disaster in older adults. Alternatively, the physical activity was not significantly different when adjusted for baseline BMI. This shows that exercise habits are an important preventive factor for undernutrition among older adults. The interaction with exercise habits was significant only at baseline BMI. Thus, to prevent undernutrition among the elderly after a disaster, it may be necessary to consider the possibility that some elderly people, without an exercise habit to begin with, could be at high risk of undernutrition, regardless of their sex, age, drinking and smoking status, and medical history.

After adjusting for BMI, the association between undernutrition and physical activity disappeared, and only exercise habits were associated with the occurrence of undernutrition. This result proposes that it is important to maintain muscle mass through more intense exercise, rather than physical activity as an extension of daily activities, to prevent undernutrition.

On the other hand, although physical activity was not associated with the occurrence of undernutrition after adjusting for BMI, there is still a possibility that physical activity before the disaster may work to prevent undernutrition. A systematic review found that physical activity promotes appetite control and balanced energy intake [30]. It also reports that physical activity is the least expensive day drug therapy because enhanced physical activity lowers the depression risk and shows a preventive effect [31]. In addition, although undernutrition is one of the risks for decline in life functions in the elderly, it has been reported that increasing the amount of daily physical activity can decrease the risk of age-related decline in life functions, including reduced motor function and dementia, and that people who practice a physically active lifestyle can live longer independently by following a physically active lifestyle [29,32,33,34]. Additionally, exercise habits have been associated with enhanced quality of life and decreased risk of upper respiratory tract infections in older adults. Interventions such as improving walking speed and increasing physical activity improve frailty [35,36,37]. Thus, we believe that having exercise habits and physical activity in daily life is essential for elderly people who are vulnerable to disasters, to reduce the risk of undernutrition caused by the decline in physical function and to improve their QOL, although the mechanisms and the range of effects are different.

### 4.2. Relationship between Exercise Habits and BMI

In this study, the analysis results, with the occurrence of undernutrition as the outcome, differed from those with the change in BMI as the dependent variable. This result indicates that the effect of exercise habits and physical activity varies depending on the BMI level of the baseline. In fact, as shown in Table 3, when the BMI was analyzed in two groups, exercise habit and physical activity were not associated with undernutrition development in the group with higher-than-average BMI, whereas, they were associated with the development of undernutrition in the group with lower-than-average BMI. In other words, exercise habits and physical activity may not be involved in weight maintenance in all people but may also contribute to weight loss suppression in people who initially had a low BMI and were at high risk of emaciation.

### 4.3. Association with Evacuation

The prevalence of undernutrition was low among the evacuees after the disaster. According to the FHMS for the same evacuated area residents, a large proportion of the people from the evacuation areas gained weight after the disaster [12,13,14,15,16,17]. The study results show that, in the evacuation areas, even older adults gained weight. However, a certain number of people in the evacuation areas were undernourished. After the disaster, life in the shelters was prolonged, with meals high in carbohydrates [14]. The FHMS reported that living in non-house conditions after the disaster was associated with a poor dietary intake of fruits and vegetables, meat, and soybean, as well as dairy products [18]. A study conducted in Miyagi Prefecture after the GEJE also proposed that while the provision of boxed lunches may supplement the calories and protein that are often lacking in evacuation centers, it may also be insufficient in providing vitamins and vegetables, proposing that there are limits to the nutrients that can be provided using boxed lunches alone [11,38]. Thus, in an environment where the content and form of meals cannot be selected, the evacuees had to eat the available food, regardless of their nutritional balance. The evacuees were therefore likely to gain weight. However, the nutritional intake might decrease in older adults with poor gastrointestinal or swallowing/chewing function. One of the problems was that the disaster management teams tended to focus on gaining weight and may have delayed their response to undernutrition. In situations such as disasters, it is essential not to miss such cases of undernutrition.

### 4.4. Late Meals

The tendency to eat meals before going to bed fewer than three times a week was a risk factor for undernutrition. Eating within two hours of bedtime is, however, regarded as a dietary habit that promotes obesity due to fat accumulation that results from insulin secretion and action of the appetite hormone leptin. In fact, eating late is known to be an unhealthy eating habit that contributes to obesity and early eating and snacking, and it has been reported to be associated with visceral fat accumulation and obesity [39]. On the other hand, older people have a higher undernutrition risk and/or weight loss than the young counterparts. Therefore, delaying dinner might help prevent undernutrition and weight loss. Nevertheless, going to bed immediately after a meal is not recommended for the long term because it degrades sleep quality and strains the gastrointestinal tract. Therefore, reviewing the dietary balance and lifestyle of older adults is an important step.

### 4.5. Comparison between the Affected Areas and the Rest of Japan

Since this study was conducted on residents in the evacuation areas and does not directly compare the results with national data, the results obtained in this study on the relationship between the occurrence of undernutrition and exercise and other lifestyle habits cannot be applied to the entire country. However, it was the case that the number of obese people in the evacuation areas, who are the subject of this study, increased more rapidly after the evacuation than in the rest of Japan [12]. In other words, it is essential to note that even in the evacuation area, where the percentage of obese people is much higher than in the rest of Japan, a certain percentage of undernourished people are found, and the results of this study indicate that lifestyle habits, such as exercise, are preventively involved. Since there are more people with undernutrition than the subjects of this study in Japan, it is necessary to verify whether there is a similar association outside the evacuation area. Additionally, it is believed that the results of this study are findings that can be applied to health management of the elderly in Japan, where disasters are common.

### 4.6. Study Limitations

Firstly, since this study was conducted on residents in the evacuation area and did not directly compare the results with national data, the results obtained in this study on the relationship between undernutrition occurrence and exercise, as well as other lifestyle habits, cannot be applied to the whole of Japan. Although obesity tends to increase after a disaster, and conscious measures are being taken, it is necessary to pay attention to the problem of thinness in the elderly. The results of this study may be applied to health management of the elderly in Japan, where disasters are common. Secondly, in this study, we only dealt with health check-up data, not death data, so the mortality rates are unknown. However, it is expected that there may have been a large number of people who died at an old age who were undernourished. In this case, there is a possibility of estimating a lower frequency of undernutrition. Thirdly, high-risk undernourished adults are more likely not to have undergone a physical check-up, which may be a selection bias in the target population. It has been reported that the most common reason for not undergoing a health check-up is “because I am visiting a medical institution”, and the proportion tends to increase with age, showing a low rate of health check-ups [40,41]. When the characteristics of the 13,378 people who could be followed in this study were compared with those of the 4244 people who could not be followed, the average age was 68.4 vs. 73.3 years, and the subjects who could not be followed were older, so the above hypothesis is possible. The possibility that there is a large percentage of healthy people among those who receive medical check-ups cannot be denied. Consequently, the overall results may be biased toward health, and the percentage of people with a tendency toward undernutrition may be underestimated. It is believed that the effects of exercise habits and physical activity would be more important even if the subjects of this study were a healthier population among the elderly. There was no difference in the distribution of sex, exercise habits, or physical activity in this comparison. Fourth, the undernutrition in certain cases was attributable to other diseases (other than lifestyle diseases); however, those patients from our analysis who might have affected the results could not be ruled out. Fifth, this study employed items from health check-ups. Therefore, there was a lack of detailed information on nutrient intake, which is strongly associated with weight loss and undernutrition. However, these present results were obtained, although only information on eating behavior was used, which suggests that the influence of diet may be underestimated but not overestimated. Sixth, the estimated weight loss (a risk factor for older adults) was 3–5 kg or 5% reduction in body weight over the previous six months to one year [25]. However, the follow-up period differed among the participants, making it challenging to determine how much weight loss occurred and over what period. Lastly, the effects of exercise habits were examined before the disaster (baseline), but the alternations changes in exercise habits after the disaster were not assessed. Therefore, whether the continuation of exercise habits affected the onset of undernutrition could not be determined. It will be necessary to examine, in the future, whether the continuation of exercise habits and physical activity is associated with preventing undernutrition occurrence.

## 5. Conclusions

The associations between the prevalence of undernutrition and related lifestyle factors after the disaster were examined using health check-up data for residents aged 60 years and above living in the evacuation areas before the disaster. The results suggested that the evacuation itself is associated with the post-disaster undernutrition occurrence, and those that have exercise habits and physical activity before the disaster may prevent post-disaster undernutrition, regardless of other lifestyle habits and gender.

## Figures and Tables

**Table 1:** Characteristics at baseline of 13,378 participants with and without incident of undernutrition.

			Total		Undernutrition		Non-Undernutrition		*p* Values *
Participants		*n*	13,378		1712	(12.8)	11,666	(87.2)	
Follow-up period, (years)		13,378	6.9	(2.2)	4.9	(2.0)	7.2	(2.1)	<0.001
Evacuee, (%)		13,378	4586	(34.3)	489	(28.6)	4097	(35.1)	<0.001
Sex(men), *n*(%)		13,378	6351	(47.5)	631	(36.9)	5720	(49.0)	<0.001
Age, (years)		13,378	68.4	(6.2)	68.8	(6.3)	68.3	(6.2)	0.003
75 years old or older, *n* (%)		13,378	2796	(20.9)	389	(22.7)	2407	(20.6)	0.047
80 years old or older, *n* (%)		13,378	659	(4.9)	99	(5.8)	560	(4.8)	0.08
Body weight, (kg)		13,378	54.7	(6.8)	50.7	(6.0)	55.2	(6.7)	<0.001
Body mass index, (kg/m^2^)		13,378	22.7	(1.3)	21.3	(1.0)	22.9	(1.3)	<0.001
Amount of weight loss, (kg)		13,378	0.53	(3.8)	4.65	(3.0)	−0.07	(3.5)	<0.001
Rate of weight loss, (%)		13,378	1.01	(7.0)	9.02	(5.4)	−0.16	(6.4)	<0.001
Rate of weight loss, (kg/year)		13,378	0.12	(0.7)	1.05	(0.8)	−0.02	(0.6)	<0.001
Rate of weight loss, (%/year)		13,378	0.22	(1.4)	2.06	(1.6)	−0.05	(1.1)	<0.001
Weight loss ≥ 5 kg, *n* (%)		13,378	1422	(10.6)	645	(37.7)	777	(6.7)	<0.001
Weight loss ≥ 5 %, *n* (%)		13,378	3560	(26.6)	1268	(74.1)	2292	(19.7)	<0.001
Exercise habits, *n* (%)	<30 min/2times/week	12,488	8061	(64.6)	1065	(67.0)	6996	(64.2)	0.03
Physical activities, *n* (%)	<1 h/day	12,495	7556	(60.5)	1010	(63.5)	6546	(60.0)	0.01
Walking speed, *n* (%)	fast	12,490	5543	(44.4)	693	(43.6)	4850	(44.5)	0.51
Insufficient sleep, *n* (%)	yes	12,532	9768	(77.9)	1212	(76.1)	8556	(78.2)	0.06
Weight change from age 20, *n* (%)	≥10 kg	12,493	2760	(22.1)	161	(10.1)	2599	(23.8)	<0.001
Weight change in 1 year, *n* (%)	≥±3 kg	12,492	2055	(16.5)	201	(12.7)	1854	(17.0)	<0.001
Meals before going to bed, *n* (%)	≥3 times/week	12,498	2835	(22.7)	294	(18.5)	2541	(23.3)	<0.001
Snack after dinner, *n* (%)	≥3 times/week	12,514	913	(7.3)	116	(7.3)	797	(7.3)	0.98
Lack of breakfast, *n* (%)	≥3 times/week	12,502	445	(3.6)	60	(3.8)	385	(3.5)	0.61
Smoking status, *n* (%)	Current smoker	13,378	1762	(13.2)	194	(11.3)	1568	(13.4)	0.02
Drinking status, *n* (%)	Non-drinker	13,378	7553	(56.5)	1098	(64.1)	6455	(55.3)	<0.001
	Current drinker, <44 g/day		5164	(38.6)	547	(32.0)	4617	(39.6)	
	Current drinker, <44 g/day		661	(4.9)	67	(3.9)	594	(5.1)	
Digestive surgery, *n* (%)	yes	13,378	821	(6.1)	120	(7.0)	701	(6.0)	0.11
Lifestyle-related diseases, *n* (%)	yes	13,378	5586	(41.8)	797	(46.6)	4789	(41.1)	<0.001
Subjective symptoms, *n* (%)	nothing	13,378	10,543	(78.8)	1339	(78.2)	9204	(78.9)	0.04
	1 symptom		2077	(15.5)	254	(14.8)	1823	(15.6)	
	2 or more		758	(5.7)	119	(7.0)	639	(5.5)	

Note. Undernutrition; BMI ≤ 20.0 kg/m^2^, non-undernutrition; BMI > 20.0 kg/m^2^. The value expressed as mean (standard deviation) or number of people (proportion). * For categorical variables, we used the χ^2^ test, and Fisher’s exact test was used, and *t*-test was used for continuous variables.

**Table 2:** Hazard ratios (95% confidence intervals) for the incidence of post-disaster undernutrition for lifestyle and sociodemographic factors among 13,378 participants.

		Sex-Age-Adjustment		Multivariable Adjustment (Model 1) *		Multivariable Adjustment (Model 2) *2		Multivariable Adjustment (Model 3) *3		Multivariable Adjustment (Model 4) *4	
Factor	Reference	HR (95% CI)	*P* Values	HR (95% CI)	*p* Values	HR (95% CI)	*P* Values	HR (95% CI)	*p* Values	HR (95% CI)	*p* Values
Sex (Women)	Men	1.63 (1.48–1.80)	<0.001	1.64 (1.45–1.85)	<0.001	1.63 (1.44–1.84)	<0.001	1.42 (1.26–1.61)	<0.001	1.42 (1.25–1.61)	<0.001
Age	1 SD (6.2 years)	1.24 (1.18–1.31)	<0.001	1.25 (1.18–1.32)	<0.001	1.24 (1.18–1.31)	<0.001	1.23 (1.17–1.30)	<0.001	1.23 (1.17–1.25)	<0.001
Age (≥75 years old)	<75 years old	1.60 (1.43–1.79)	<0.001								
Age (≥80 years old)	<80 years old	1.96 (1.60–2.41)	<0.001								
BMI at baseline	−1 SD (1.35 kg/m^2^)	0.24 (0.23–0.26)	<0.001					0.24 (0.23–0.26)	<0.001	0.24 (0.23–0.26)	<0.001
Evacuation (no)	Yes	1.44 (1.29–1.60)	<0.001	1.40 (1.26–1.56)	<0.001	1.40 (1.26–1.55)	<0.001	1.38 (0.12–1.54)	<0.001	1.39 (1.25–1.54)	<0.001
Walking speed (fast)	Slow	0.94 (0.85–1.03)	0.18								
Insufficient sleep (yes)	No	1.11 (0.99–1.25)	0.08								
Exercise habits (<30 min/2 times/week)	≥30 min/2times/week	1.16 (1.04–1.29)	0.006	1.14 (1.03–1.27)	0.02			1.11 (1.00–1.24)			
Physical activities (<1 h/day)	≥1 h/day	1.15 (1.04–1.27)	0.009			1.12 (1.01–1.25)	0.03			1.08 (0.98–1.20)	0.14
Smoking status (yes)	No	1.20 (1.02–1.41)	0.03	1.16 (0.99–1.37)	0.07	1.16 (0.99–1.37)	0.07	1.01 (0.86–1.19)	0.93	1.01 (0.85–1.18)	0.95
Drinking status (<44 g/day)	Non-drinker	0.87 (0.77–0.97)	0.02	0.89 (0.79–1.00)	0.05	0.89 (0.79–1.00)	0.04	0.90 (0.79–1.01)	0.07	0.89 (0.79–1.00)	0.06
(≥44 g/day)	Non-drinker	1.03 (0.79–1.34)	0.86	1.07 (0.82–1.39)	0.64	1.06 (0.81–1.39)	0.66	0.96 (0.73–1.25)	0.75	0.95 (0.73–1.25)	0.71
Meals before going to bed (<3 times/week)	≥3 times/week	1.27 (1.12–1.44)	<0.001	1.26 (1.11–1.43)	<0.001	1.25 (1.10–1.42)	<0.001	1.19 (1.05–1.35)	<0.01	1.18 (1.04–1.35)	<0.01
Snack after dinner (≥3 time/week)	<3 times/week	1.01 (0.84–1.22)	0.90								
Digestive surgery (yes)	No	1.27 (1.05–1.53)	0.01	1.24 (1.03–1.50)	0.02	1.24 (1.02–1.49)	0.03	1.02 (0.84–1.23)	0.85	1.02 (0.85–1.23)	0.83
Lifestyle-related diseases (yes)	No	1.29 (1.17–1.42)	<0.001	1.27 (1.16–1.40)	<0.001	1.28 (1.16–1.41)	<0.001	1.02 (0.92–1.12)	0.76	1.02 (0.93–1.12)	0.69
Subjective symptoms (1 symptom)	No symptoms	0.96 (0.84–1.10)	0.54	0.98 (0.86–1.13)	0.85	0.98 (0.86–1.13)	0.86	0.996 (0.87–1.14)	0.95	0.997(0.87–1.14)	0.96
(2 or more symptoms)	No symptoms	1.25 (1.04–1.51)	0.02	1.26 (1.04–1.53)	0.02	1.26 (1.04–1.52)	0.02	1.36 (1.12–1.64)	<0.01	1.36 (1.12–1.64)	<0.01

Note. HR: hazard ratio; CI: confidence interval; SD: standard deviation. Dependent variable: undernutrition. Independent variable of interest: exercise habits or physical activity. * Model 1: Adjustment variables included in the model: age (continuous variable), sex, evacuation, exercise habits, smoking status, drinking status, meals before going to bed, digestive surgery, lifestyle-related diseases, and subjective symptoms. *2 Model 2: Adjustment variables included in the model: age (continuous variable), sex, evacuation, physical activity, smoking status, drinking status, meals before going to bed, digestive surgery, lifestyle-related diseases, and subjective symptoms. *3 Model 3: Adjustment variables included in the model: age (continuous variable), sex, BMI (at baseline), evacuation, exercise habits, smoking status, drinking status, meals before going to bed, digestive surgery, lifestyle-related diseases, and subjective symptoms. *4 Model 4: Adjustment variables included in the model: age (continuous variable), sex, BMI (at baseline), evacuation, physical activity, smoking status, drinking status, meals before going to bed, digestive surgery, lifestyle-related diseases, and subjective symptoms.

**Table 3:** Hazard ratios (95% CIs) of exercise habits for undernutrition incidence after disaster among 13,378 participants are stratified by each lifestyle and sociodemographic factor.

		Exercise Habit				Physical Activity			
		≥30 min/2times/Week	<30 min/2times/Week			≥1 h/Day	<1 h/Day		
Number of participants		4427	8061			4939	7556		
Number of undernutrition		525	1065			580	1010		
follow-up years		7.09	6.98			7.02	7.01		
Total person years		31,381	56,249			34,678	53,004		
Incidence rate of undernutrition (1000 person years)		16.7	18.9			16.7	19.1		
			HR (95% CI)	*p* values *1	*p* for interaction		HR (95% CI)	*p* values *2	*p* for interaction
Sex	Men	Reference	1.21 (1.02–1.44)	0.03	0.48	Reference	1.08 (0.92–1.28)	0.34	0.42
	Women	Reference	1.10 (0.96–1.25)	0.18		Reference	1.15 (1.01–1.31)	0.04	
Age group	≥68 years old	Reference	1.05 (0.90–1.22)	0.54	0.98	Reference	1.07 (0.92–1.24)	0.40	0.87
	<68 years old	Reference	1.22 (1.05–1.42)	<0.001		Reference	1.16 (1.01–1.34)	0.04	
BMI	≥22.7	Reference	0.98 (0.73–1.32)	0.91	<0.001	Reference	1.04 (0.77–1.39)	0.81	0.87
	<22.7	Reference	1.15 (1.03–1.29)	0.02		Reference	1.11 (0.99–1.24)	0.07	
Evacuation	No	Reference	1.19 (1.05–1.35)	0.01	0.29	Reference	1.10 (0.97–1.24)	0.13	0.37
	Yes	Reference	1.02 (0.85–1.24)	0.81		Reference	1.18 (0.98–1.43)	0.09	
Smoking status	No	Reference	1.10 (0.98–1.23)	0.09	0.08	Reference	1.14 (1.02–1.27)	0.02	0.33
	Yes	Reference	1.54 (1.10–2.14)	0.01		Reference	0.98 (0.73–1.32)	0.90	
Drinking status	Non-drinker	Reference	1.09 (0.95–1.24)	0.22		Reference	1.19 (1.05–1.36)	0.01	
	<44 g/day	Reference	1.22 (1.02–1.46)	0.03	0.38	Reference	1.04 (0.87–1.25)	0.65	0.34
	≥44 g/day	Reference	1.46 (0.84–2.54)	0.18	0.35	Reference	0.98 (0.59–1.62)	0.93	0.56
Meals before going to bed	<3 times/week	Reference	1.16 (0.90–1.47)	0.25	0.67	Reference	1.19 (0.94–1.51)	0.15	0.49
	≥3 times/week	Reference	1.14 (1.02–1.28)	0.03		Reference	1.11 (0.99–1.25)	0.07	

Note. *1 Adjustment variables included in the model: age (continuous variable), sex, evacuation, exercise habits, smoking status, drinking status, meals before going to bed, digestive surgery, lifestyle-related diseases, and subjective symptoms. *2 Adjustment variables included in the model: age (continuous variable), sex, evacuation, physical activity, smoking status, drinking status, meals before going to bed, digestive surgery, lifestyle-related diseases, and subjective symptoms.

## Data Availability

The datasets analyzed during the present study are not publicly available because the data from the Fukushima Health Management Survey belongs to the government of Fukushima Prefecture and can only be used within the organization.

## Supplementary Materials

The following supporting information can be downloaded at https://www.mdpi.com/article/10.3390/ijerph19063399/s1. Table S1: Multiple regression analysis with the change in BMI from baseline as the dependent variable; Table S2: Comparison of the average change in BMI for each factor such as lifestyle factors.
