# Supplementary material for: Lifestyle Factors Associated with Undernutrition in Older People after the Great East Japan Earthquake: A Prospective Study in the Fukushima Health Management Survey
Source: Int J Environ Res Public Health. 2022 Mar 14;19(6):3399. doi: 10.3390/ijerph19063399 (PMC8951088; doi:10.3390/ijerph19063399)
Supplement: Supplementary file 1 [file ijerph-19-03399-s001.zip › ijerph-1553759-supplementary.pdf]

Supplemental Table S1. Multiple regression analysis with the change in BMI from baseline as the dependent variable

		**Multivariable adjustment (Model1) ***		**Multivariable adjustment (Model2) ****	
**Factor**	**Reference**	Standardized β	p values	Standardized β	p values
Sex (women)	Men	–0.110	<0.001	–0.110	<0.01
Age	1SD (6.2 years)	–0.108	<0.001	–0.110	<0.001
Evacuation (no)	Yes	–0.466	<0.001	–0.466	<0.001
Exercise habits (<30min/2times/week)	≥30min/2times/week	0.020	0.49		
Physical activities (<1hour/day)	≥1hour/day			0.004	0.89
Smoking status (yes)	No	0.240	<0.001	0.240	<0.001
Drinking status (< 44g/day)	Non-drinker	0.022	0.50	0.021	0.52
(≥ 44g/day)	Non-drinker	0.029	0.67	0.029	0.67
Meals before going to bed (< 3times/week)	≥3times/week	–0.164	<0.001	-0.165	<0.001
Digestive surgery (yes)	No	–0.101	0.08	–0.102	0.08
Lifestyle-related diseases (yes)	No	0.020	0.48	0.020	0.47
Subjective symptoms (1 symptom)	No symptom	–0.052	0.17	–0.051	0.18
(2 or more symptoms)	No symptom	–0.066	0.27	–0.065	0.27

Dependent variable: change in BMI from baseline. Independent variable of interest: exercise habits or physical activity.

* Model1 : Adjustment variables included in the model: age(continuous variable), sex, Evacuation, exercise habits, smoking status, drinking status, meals before going to bed, digestive surgery, lifestyle-related diseases, and subjective symptoms.

** Model2 : Adjustment variables included in the model: age(continuous variable), sex, evacuation, physical activity, smoking status, drinking status, meals before going to bed, digestive surgery, lifestyle-related diseases, and subjective symptoms.

Supplemental Table S2. Comparison of the average change in BMI for each factor such as lifestyle factors

		N	Mean	(SD)	P
Evacuation	Yes	4,586	0.399	(1.78)	<0.001
	No	8,793	-0.060	(1.47)	
Sex	Men	6,352	0.182	(1.53)	<0.001
	Women	7,027	0.021	(1.65)	
Age	>68	6,025	0.005	(1.63)	<0.001
	<68	7,354	0.173	(1.56)	
BMI <20kg/m^2	No	11,667	0.378	(1.44)	<0.001
	Yes	1,712	-1.812	(1.22)	
BMI at baseline	<Mean	6,483	0.154	(1.54)	<0.001
	>Mean	6,896	0.045	(1.64)	
Exercise habits	Enough	4,427	0.091	(1.58)	0.3787
	Poor	8,062	0.117	(1.60)	
Physical activities	Enough	4,940	0.112	(1.60)	0.7773
	Poor	7,556	0.104	(1.59)	
Smoking status	No	11,617	0.054	(1.58)	<0.001
	Yes	1,762	0.382	(1.66)	
Drinking status	No	7,553	0.037	(1.64)	<0.001
	<44g/day	5,165	0.160	(1.54)	
	>44g/day	661	0.300	(1.51)	
Meals before going to bed	No	9,664	0.063	(1.58)	<0.001
	Yes	2,835	0.252	(1.64)	
Digestive surgery	No	12,558	0.105	(1.60)	0.0404
	Yes	821	-0.013	(1.52)	
Lifestyle-related diseases	No	7,792	0.077	(1.61)	0.0801
	Yes	5,587	0.126	(1.57)	
Subjective symptoms	No	10,544	0.110	(0.11)	0.1978
	1	2,077	0.054	(1.66)	
	>1	758	0.040	(1.69)	
